# Harnessing a High Cargo-Capacity Transposon for Genetic Applications in Vertebrates

**DOI:** 10.1371/journal.pgen.0020169

**Published:** 2006-11-10

**Authors:** Darius Balciunas, Kirk J Wangensteen, Andrew Wilber, Jason Bell, Aron Geurts, Sridhar Sivasubbu, Xin Wang, Perry B Hackett, David A Largaespada, R. Scott McIvor, Stephen C Ekker

**Affiliations:** 1 The Arnold and Mabel Beckman Center for Transposon Research, Institute of Human Genetics, University of Minnesota, Minneapolis, Minnesota, United States of America; 2 Department of Genetics, Cell Biology, and Development, University of Minnesota, Minneapolis, Minnesota, United States of America; 3 Department of Biochemistry, Molecular Biology, and Biophysics, University of Minnesota, Minneapolis, Minnesota, United States of America; 4 Gene Therapy Program, University of Minnesota, Minneapolis, Minnesota, United States of America; 5 Cancer Center, University of Minnesota, Minneapolis, Minnesota, United States of America; 6 Stem Cell Institute, University of Minnesota, Minneapolis, Minnesota, United States of America; RIKEN Genomic Sciences Center, Japan

## Abstract

Viruses and transposons are efficient tools for permanently delivering foreign DNA into vertebrate genomes but exhibit diminished activity when cargo exceeds 8 kilobases (kb). This size restriction limits their molecular genetic and biotechnological utility, such as numerous therapeutically relevant genes that exceed 8 kb in size. Furthermore, a greater payload capacity vector would accommodate more sophisticated *cis* cargo designs to modulate the expression and mutagenic risk of these molecular therapeutics. We show that the *Tol2* transposon can efficiently integrate DNA sequences larger than 10 kb into human cells. We characterize minimal sequences necessary for transposition *(miniTol2)* in vivo in zebrafish and in vitro in human cells. Both the 8.5-kb *Tol2* transposon and 5.8-kb *miniTol2* engineered elements readily function to revert the deficiency of fumarylacetoacetate hydrolase in an animal model of hereditary tyrosinemia type 1. Together, *Tol2* provides a novel nonviral vector for the delivery of large genetic payloads for gene therapy and other transgenic applications.

## Synopsis

Mobile genetic elements (transposons) are effective vehicles for the delivery of foreign DNA for gene therapy and gene discovery applications. Their utility in vertebrates has been, however, limited to relatively few known elements with high activity, including the engineered element *Sleeping Beauty (SB)* and the naturally occurring fish transposon, *Tol2*. The authors explore and systematically unlock some of the potential of *Tol2*, characterizing a minimal set of transposon sequences required for gene transfer by the *Tol2*-encoding enzyme, transposase. The authors further demonstrate full activity of this “mini” element in human tissue culture cells and in the treatment of a mouse model of tyrosinemia. *Tol2* demonstrates high cargo-capacity, readily transferring large (at least 10,000 base pairs) DNA sequences, an ability that opens the door to an array of molecular genetic approaches in vertebrates previously difficult or impossible using prior tools.

## Introduction

The natural medaka fish hAT gene family element *Tol2* [1–3], the engineered Tc1/*mariner* transposons *Sleeping Beauty (SB)* [4] and *Frog Prince* [5], and the insect-derived natural element *PiggyBac* [6] represent transposons potentially suitable as DNA transfer tools for gene discovery and gene delivery applications in vertebrates. During the past decade, we have been studying the *Sleeping Beauty* transposable element for molecular genetic applications in vertebrates. While exceptionally active in higher vertebrates [7], the SB system does have two significant shortcomings: relatively modest cargo-capacity and decreased activity under high transposase concentrations *(overexpression inhibition)* [8,9]. The availability of an alternative, active transposon system devoid of these disadvantages and adapted for use in higher vertebrates would offer tremendous potential uses in a variety of molecular genetic and biotechnological fields. There is also a potential for synergism between multiple transposon systems, both in gene discovery and in gene transfer applications. *Tol2* is widely used as a transgenesis tool in the zebrafish Danio rerio due to its high level of activity in this organism's germline [10,11]. However, gene transfer into cultured human cells or live mammals using *Tol2* has not been previously reported, nor have the transposon sequence requirements for *Tol2*-based transposition been previously well characterized.

## Results

While testing green fluorescent protein (GFP)-marked *Tol2* vectors in zebrafish, we noticed an unusually high number of GFP-positive cells in embryos injected with *Tol2* transposon and synthetic transposase mRNA derived from an updated *Tol2* transcription vector (Figure 1), suggesting that this transposon is highly active in somatic tissues. The difference was particularly striking (over 10-fold) when percentages of embryos exhibiting eye fluorescence were evaluated (Figure 1A, right panel, red arrows). This effect was not observed when a *Sleeping Beauty* transposon with the same expression cassette was previously used in zebrafish (Figure 1 and [12,13]). To determine if this increase in overall and particularly eye fluorescence was a result of increased transposition, we conducted a PCR-based assay for one of the molecular markers of the transposition reaction—generation of a transposon-excision footprint [14]. We found that increased GFP fluorescence in *Tol2*-injected embryos versus *SB*-injected embryos correlated with transposon excision. The *Tol2* excision band was more robust than the *SB* excision band and appeared much earlier, as soon as 2 h after injection (Figure 1B). This may reflect a kinetic difference between the two systems; *SB* is thought to function as a tetramer [4,15] and may take longer to form an active complex than *Tol2,* which may function like another hAT family member *Hermes,* i.e., as a dimer [16]. We also note that the high *Tol2* somatic transposition rate correlates with germline transposition: 18 of 25 tested fish gave GFP-positive progeny (a 72% transgenesis and expression rate), with an average founder fish transmitting an estimated five independent insertion events (unpublished data).

**Figure 1:**
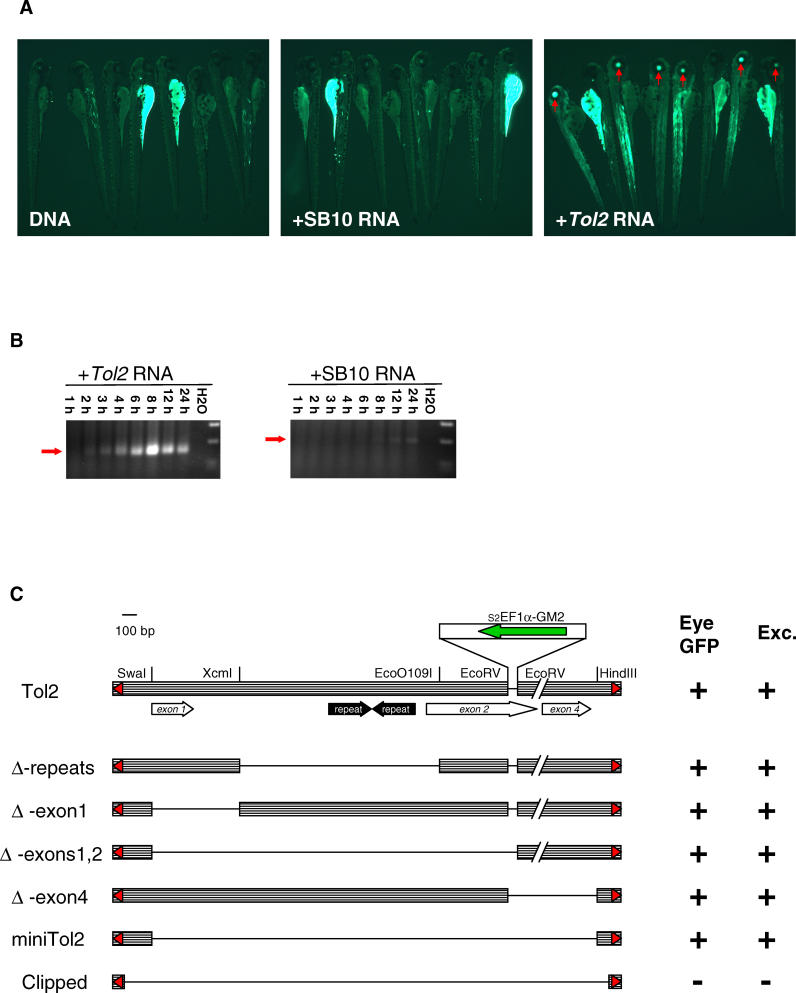
Functional Characterization of *Tol2* Transposon Sequences Using a Somatic Transposition Assay in Zebrafish (A) Visualization of *Tol2*-mediated somatic transposition in zebrafish (see somatic transposition assay in Materials and Methods). Left, Injection of transposon DNA alone results in a highly mosaic expression pattern with fewer than 5% of the animals displaying eye expression [13,50]. Middle, Injection of *SB* transposase RNA does not significantly alter this somatic gene transfer distribution [12]. Right, Injection of *Tol2* transposase RNA results in gene transfer into the larval eye with over 75% of the injected embryos displaying gene transfer in this tissue at 3 dpf (red arrows). (B) Molecular evidence of rapid *Tol2*-transposase activity in injected zebrafish embryos. Time-course analysis of excision from injected plasmid DNA [14] was conducted on animals co-injected with a mixture of GFP-marked *Tol2* (pTol2/S2EF1a-GM2) and *SB* (pT2/S2EF1a-GM2 [13] transposons with either *Tol2* or *SB* RNA. Note the kinetic delay in activity maturation by the presumptive obligate tetrameric *SB* transposase compared to *Tol2* (see text). Red arrows indicate the resulting transposase-dependent excision product. (C) Deletion analysis identifies minimal *Tol2* sequences required for gene transfer and transposon excision in zebrafish. Zebrafish embryos were injected with depicted deletion constructs and transposase RNA and scored for GFP fluorescence at 3 d postfertilization (eye GFP column). Twenty GFP-positive embryos were used to prepare DNA for excision PCR (Exc. Column). Striped boxes represent *Tol2* transposon sequences, with red triangles indicating terminal inverted repeats. Structural elements present in the commonly used *Tol2* vector are depicted, with exons shown as open arrows and internal inverted repeats as solid arrows. Restriction enzyme sites are indicated above the transposon drawing.

This increase in GFP fluorescence together with generation of a robust excision footprint provides a rapid *Tol2 *activity assay using zebrafish embryos. We used this assay to address a significant drawback of *Tol2*—minimal sequences required for transposition had not been previously defined. The commonly used *Tol2* vector [11,17] has deletions encompassing part of exon-2, all of exon-3, and part of exon-4 with intervening introns. While these deletions render the transposase inactive, they leave intact a significant fraction of the transposase open reading frame with several splice sites, as well as promoter and polyadenylation sequences. The presence of these sequences hinders vector design and compromises vector utility in its application to gene discovery, gene transfer, and gene therapy. For gene therapy applications in particular, they pose an increased risk of insertional mutagenesis. We made a systematic series of deletions of the *Tol2* vector and tested them in our zebrafish somatic transposition assay (Figure 1C). Surprisingly, the first four deletions were all active in this assay, even though two of them removed sequences previously thought to be required for transposon excision [18]. A combination of 5′ and 3′ deletions resulted in the smallest active element containing 261 nucleotides of 5′ *Tol2* DNA and 202 nucleotides of *Tol2* 3′ DNA (“*miniTol2*”). A further truncation of the *Tol2* sequences (“*Tol2* clipped”) by PCR inactivated the element, however, indicating that there are sequence requirements beyond the minimal 17-base inverted terminal repeats. The *miniTol2* vector was subsequently tested in human cells in vitro (Figure 2) and for gene transfer in vivo (Figure 3) and demonstrated activity statistically indistinguishable from that of the full-length vector in each case.

**Figure 2:**
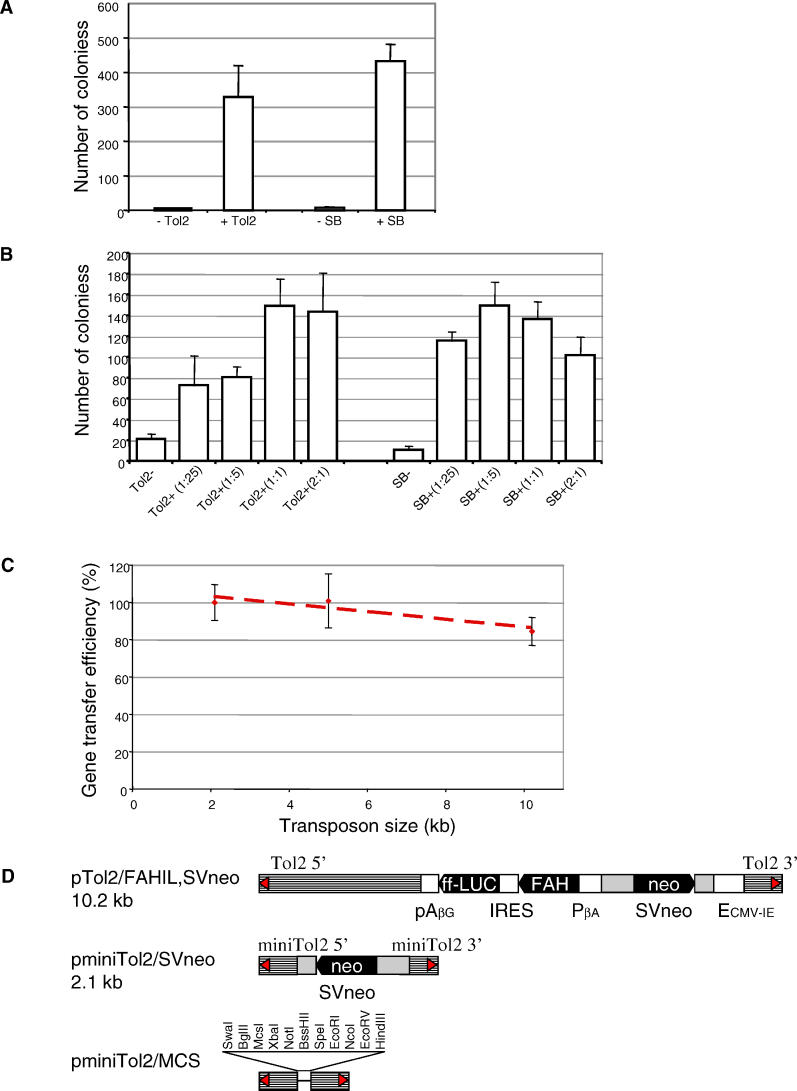
Large Cargo-Capacity, *Tol2*-mediated Transposition in Human Cells (A) Comparison of *Tol2* and *SB* transposons in G418-resistant colony-forming assay in HeLa cells. *SB* (pT2/SVneo) and *Tol2* (pTol2/SVneo) transposons with corresponding transposase-coding plasmids (pCMV-SB10 or pCMV-Tol2) were transfected into HeLa cells under conditions optimized for *SB* [8]. Transposon plasmid amounts were adjusted to be in equal molar ratio to 500 ng of pT2/SVNeo and the transposase construct amounts were based on 500 ng of pCMV-SB10. For negative control, a CMV-driven GFP plasmid (pGL-1) was used instead of transposase. Three independent preparations of each transposon plasmid were used in each experiment. (B) *Tol2*-mediated gene transfer activity is a positive function of *Tol2* transposase activity. *SB* (pT2/SVneo [8]) and *Tol2* (pTol2/SVneo depicted below the graph) transposons were transfected into HT1080 cells at various ratios of transposon to transposase (pCMV-SB10 [8] and pCMV-Tol2, respectively). For control experiments, pCMV-GFP plasmid was used. Total number of G418-resistant colonies per plate is plotted on the *y*-axis with error bars representing standard error of the mean of three transfection plates. In the transposon depiction below the graph, *Tol2* sequences are shown as striped boxes with terminal inverted repeats indicated by red triangles. (C) *Tol2* gene transfer efficiencies as a function of cargo-capacity. *x*-axis, transposon size in kilobases; *y*-axis, relative efficiency. *Tol2* transposons of different sizes (2.1 kb, miniTol2/SVneo; 5.0 kb, Tol2/SVneo; 10.2 kb, Tol2/SVneo,FAHIL; two of them depicted below the graph) were transfected into HT1080 cells and selected for G418 resistance. The number of colonies obtained using each transposon was determined, and relative efficiency was calculated as percentage activity compared to miniTol2/SVneo ± standard error (*n* = 3). Red line represents a linear interpolation of activity as a function of *Tol2* transposon size. (D) *Tol2* transposon constructs. Top, pTol2/FAHIL,SVneo. ECMV-IE is CMV immediate-early enhancer (open box), PβG is the chicken beta globin promoter (open box), pAβG is rabbit beta globin polyA, and IRES is encephalomyocarditis virus internal ribosome entry site (open box). Middle, pminiTol2/SVneo. Bottom depiction, *miniTol2* vector with multiple cloning site (MCS) designed to facilitate use of this vector by the genetics community.

**Figure 3:**
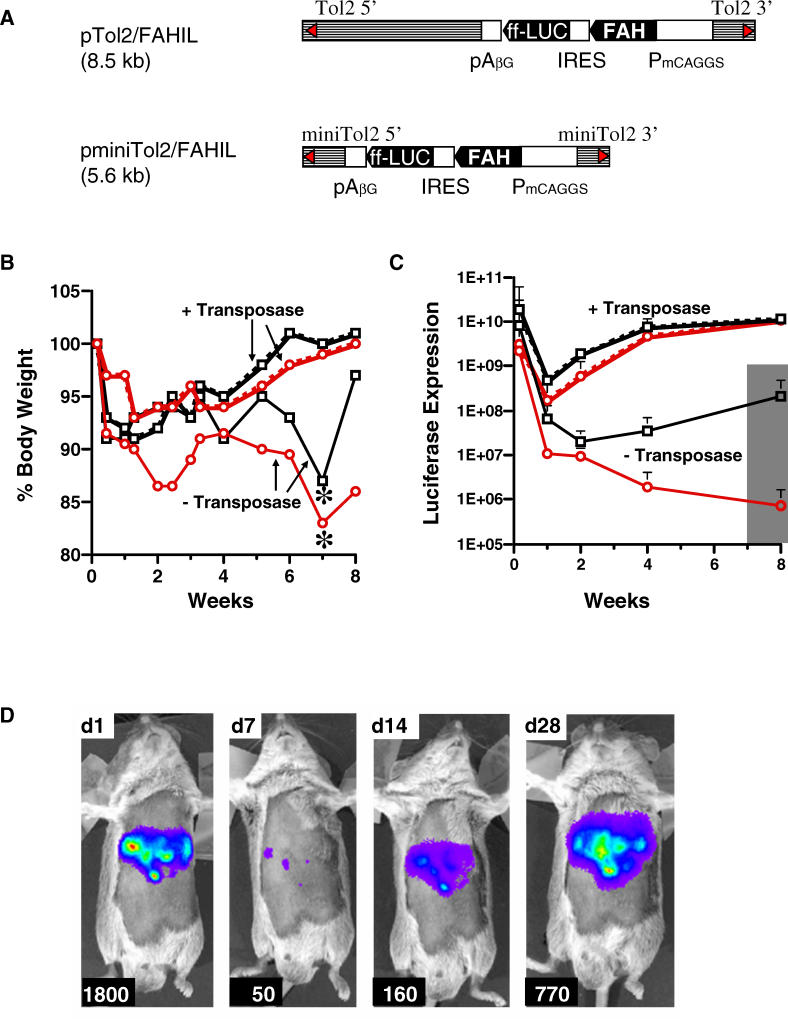
*Tol2*-Mediated, Long-term Expression and Gene Correction of Murine Model of Tyrosinemia Type 1 (A) Schematic diagram of *Tol2* transposons used for injections of FAH-deficient mice. The full-length (pTol2/FAHIL) and minimal (pminiTol2/FAHIL) versions of the transposon provide expression of both FAH and luciferase as products of a single message; pCMV-GFP served as a co-delivered control plasmid while pCMV-Tol2 provided a source of transposase protein. PmCAGGS [51], minimal chimeric CMV enhancer/chicken beta-actin fusion promoter; pAβG, rabbit beta-globin poly(A); ff-LUC, firefly luciferase; FAH, mouse fumarylacetoactate hydrolase cDNA; IRES, encephalomyocarditis virus internal ribosome entry site. Then 10 mg of pTol2/FAHIL or pminiTol2/FAHIL transposon DNA plus pCMV-GFP or -Tol2 (10 mg each) was administered by rapid, high-volume tail vein injection into sedated FAH-deficient mice (*n* = 3 to 5 per group). (B) The mean percentage body weight for animals in each treatment group, to determine the progression of liver disease. An asterisk (*) indicates the time when NTBC was readministered in drinking water to prevent mortality of mice that did not receive Tol2 transposase. (C) In vivo luciferase activity levels, assayed at indicated time points by whole body imaging and recorded as photons emitted per second as described in Materials and MethodsMaterials and Methods. The time courses of changes in body weight or in vivo luciferase enzyme activity are shown for groups of animals co-infused with pTol2/FAHIL and GFP (squares) or *Tol2* transposase (circles), or with pminiTol2/FAHIL plus GFP (triangles) or *Tol2* transposase (plus sign). The mean percentage luciferase activity at any given time point is relative to that observed at 24 h for pminiTol2/FAHIL, which demonstrated the highest transient level of luciferase activity. The 8-wk measurement of no-transposase control animals is shaded because they required administration of NTBC for survival at 7 wk. (D) Images of mice infused with pminiTol2/FAHIL plus pCMV-Tol2 at indicated times (in days; upper left box), showing tissue regeneration as seen by increasing intensity of luciferase enzyme activity detected at a location corresponding to the liver. In vivo luciferase activity levels are reported at the bottom of each image (lower left box) as photons emitted per second (×10^6^).

In initial experiments in mammalian cells, modest gene transfer activity was noted for *Tol2* in mouse ES cells when very high amounts of transposase-encoding plasmid were used [10]. *Tol2* has also been shown to excise in HeLa and NIH/3T3 cells [19]; however, *Tol2* transposase-mediated gene transfer was notably not described in latter studies using human cells. These observations combined with high activity of *Tol2* in zebrafish somatic tissues prompted us to compare the activity of *Tol2* to that of the best-studied and highly active vertebrate transposon, *SB*. We found that in HeLa cells, the activity of 5-kb Tol element was indistinguishable from that of a 2-kb SB element (Figure 2A). We then tested the activity of Tol2 as a function of transposase concentration in HT1080 cells by varying the amount of transposase-encoding plasmid. *Sleeping Beauty* is known to exhibit decreased activity in presence of excess transposase, a phenomenon termed “overexpression inhibition” [8]. We found that the *Tol2* transposon system does not exhibit overexpression inhibition within the tested transposase concentration range, while peak gene transfer rates of the two systems are very similar despite the difference in transposon size (Figure 2B).

Restrictions in cargo-capacity (less than 8 kb) have been reported for integrating viral vectors including retroviruses and lentiviruses [20]. The *SB* transposon system exhibits a similar but less absolute capacity restriction, such that 10-kb elements are notably less mobile than 2-kb–sized *SB* transposons [8]. The larger native size of *Tol2* (5 kb) versus the presumed size of the *SB* progenitor (2 kb) might explain the surprisingly high activity that was observed for the 5-kb *Tol2* element in human cells (Figure 2A). We tested a larger, greater-than-10-kb *Tol2* element and found that it mediated gene transfer nearly as efficiently as the 5-kb (Figure 2C) element. Using a smaller, 2-kb *miniTol2* element, we found *Tol2* activity to be largely independent of reduced cargo load. *Tol2* thus exhibits an effective, high-capacity gene transfer capability in human cells.

Large cargo-capacity and gene transfer efficiency in human cells make *Tol2* an excellent candidate for molecular medicine applications. We therefore tested whether *Tol2* can be used for gene delivery in live animals. We first generated a *Tol2* transposon encoding a firefly luciferase expression cassette to facilitate in vivo imaging of live animals. We then injected 5 μg of pTol2/luc with varying amounts of pCMV-Tol2 to achieve gene transfer into mouse liver using a rapid, high-volume (hydrodynamic) injection method [21,22]. Luciferase expression was assayed by bioluminescence imaging at day 1 and subsequently followed through 28 wk. While we did not observe overexpression inhibition by using large amounts of pCMV-Tol2 (transposon:transposase plasmid ratios of up to 1:10), the minimal amount of transposase plasmid required to achieve optimal, long-term expression was with transposon:transposase plasmids at a ratio of 1:1 (unpublished data).

We next investigated the ability of *Tol2* to correct a mouse model of hereditary tyrosinemia type 1 (HT1). HT1 is caused by deficiency of fumarylacetoacetate hydrolase (FAH) leading to the accumulation of fumarylacetoacetate, which is toxic to hepatocytes. HT1 patients can be treated with 2-(2-nitro-4-trifluoro-methylbenzoyl)-1,3 cyclohexanedione (NTBC), a compound that blocks an enzyme upstream of FAH, thus preventing toxicity [23]. Mice deficient in FAH develop symptoms of HT1, providing a model to study the disease [24]. To test the effectiveness of *Tol2* in this gene therapy application, FAH knockout mice maintained on NTBC were treated by hydrodynamic injection of either standard or minimal (mini) versions of pTol2/FAHIL, a transposon that encodes both murine FAH and firefly luciferase as products of a single transcription unit (Figure 3) (A. Wilber, K. J. Wangnesteen, Y. Chen, L. Zhuo, J. L. Frandsen, J. B. Bell, Z. J. Chen, S. C. Ekker, R. S. McIvor, and X. Wang, unpublished data). One day after injection, luciferase expression was assayed by in vivo bioluminescence imaging to provide a relative assessment of gene transfer efficiency for each animal (Figure 3D). NTBC was then withdrawn to induce tyrosinemia. For subsequent images taken during the first 4 wk, all animals were administered NTBC for the 24 h preceding the time of imaging to minimize any adverse effect resulting from administration of the anesthetic, which is metabolized by the liver. Previous studies have found body weight after withdrawal of NTBC is a good measure of therapeutic effect ([25]; A. Wilber, K. J. Wangnesteen, Y. Chen, L. Zhuo, J. L. Frandsen, J. B. Bell, Z. J. Chen, S. C. Ekker, R. S. McIvor, and X. Wang, unpublished data). Therefore, body weight measurements were taken for each animal to assess therapeutic efficacy, expressed as the percentage of weight at the beginning of the experiment (Figure 3C). Animals infused with either version of pTol2/FAHIL plus pCMV-GFP required readministration of NTBC in the drinking water for 5 d (starting at the time indicated by an asterisk in Figure 3B) to survive. Importantly, animals coinjected with *Tol2* transposase-encoding plasmid did not require additional NTBC, except just prior to anesthesia for imaging, and maintained weight during the subsequent period of study, indicating that the metabolic deficiency in these animals had been corrected [25,26].

Because the pTol2/FAHIL and pminiTol2/FAHIL transposons include the luciferase-coding sequence on the same transcript as the FAH-coding sequence, we were able to evaluate the extent of liver repopulation resulting from random recombination compared to co-delivery of transposase in individual animals over time by bioluminescence imaging (Figure 3C). Emitted light was measured after luciferin injection as an indication of stable gene expression at several times over 2 mo. Figure 3C represents luciferase expression over time after injection of pTol2/FAHIL and pminiTol2/FAHIL with or without pCMV-Tol2. Figure 3D depicts the images obtained for a representative animal co-infused with pminiTol2/FAHIL plus pCMV-Tol2, showing the increase in luciferase activity observed during the course of repopulation. We observed a notable decrease in transient gene expression (day 1) for pTol2/FAHIL compared to pminiTol2/FAHIL, possibly resulting from lower rates of uptake of the larger pTol2/FAHIL plasmids compared to pminiTol2/FAHIL, which are 3.1 kb smaller. Thus, the reduction in gene transfer rate does not necessarily reflect a lower transposition rate of pTol2/FAHIL. Alternatively, sequences found within the *Tol2* transposon but not within *miniTol2* may reduce transient expression of luciferase from nonintegrated plasmid DNA. After 2 mo, FAH-deficient animals infused with pCMV-Tol2 exhibited about a 100-fold increase in luciferase expression over animals that did not receive pCMV-Tol2 (Figure 3D). In general, the kinetics of liver repopulation using either the longer version or mini derivative of the *Tol2* transposon were similar, suggesting that *Tol2* is capable of efficiently integrating transposons as large as 8.5 kb in vivo.

To confirm that the observed stable gene expression was due to transposition and not increased random integration, we used inverse PCR to recover *Tol2* insertions from mouse and human chromosomes. A total of 36 integration sites were analyzed: 24 from HT1080 cell colonies, two from HT1080 transient transfections, and ten from mouse liver (Table 1). All had hallmarks suggesting transposition; 22 of the integration sites displayed the expected 8-bp target-site duplication, while the remaining two had 7-bp target-site duplications. These results confirm that *Tol2* mediates authentic transposition in mammalian cells and tissues. We thus conclude that *miniTol2* is an effective, large cargo-capacity transposase-mediated gene transfer tool suitable for gene therapy and other vertebrate gene transfer applications.

**Table 1:**
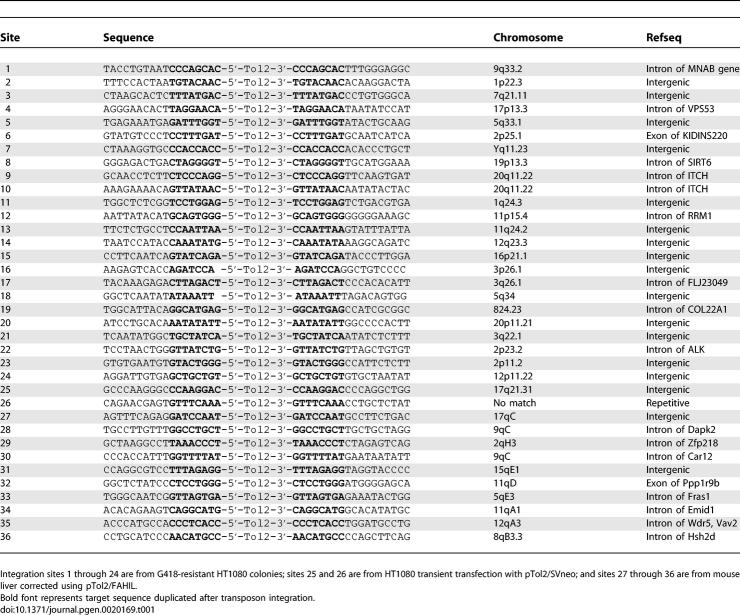
Tol2 Insertion Sites Cloned from Human HT1080 Cells and Mouse Liver pTol2/SVneo Colonies, HT1080 Cells

## Discussion

We have demonstrated that the *Tol2* transposable element from medaka fish shows high potential as a mammalian gene transfer agent. *Tol2* is highly active in human cells in vitro and in mouse liver in vivo, displaying activity comparable to that of the most active vertebrate transposon, *Sleeping Beauty*. The gene transfer rate of *Tol2* in vivo is sufficient to achieve correction of the mouse model of hereditary tyrosinemia type 1. *Tol2* exhibits two additional features that make it a very attractive gene transfer system: apparent absence of overexpression inhibition and the ability to ferry large DNA cargo. We demonstrate that a 10-kb *Tol2* transposon is nearly as active as a 2-kb element. We defined a minimized set of inverted terminal repeat sequences required for *Tol2* transposition and generated a high-utility *miniTol2* vector for gene transfer that can be used from zebrafish to clinical applications.

The ability to efficiently move cargo as large as 10-kb sets Tol2 apart from most other integrating gene transfer vectors. The best-studied vertebrate transposon system, *Sleeping Beauty*, retains only about 20% activity with such large cargo [8]. Integrating viruses, on the other hand, can only pack about 8 kb of exogenous DNA into their capsids, structurally limiting cargo-capacity [20]. Bacteriophage-derived integrases are capable of integrating such large cargo into vertebrate genomes [27,28]. Integrases tend to integrate into several pseudosites in a vertebrate genome, which can be either an advantage or a drawback depending on application. It appears that these preferred sites are not only different between species but also differ depending on the cell type used [29–32]. Recent studies demonstrate that phiC31 integrase induces chromosomal translocations in mammalian cells, which is highly undesirable for any application [32,33]. The frequency of such events needs to be thoroughly investigated to determine if this poses a significant safety risk in gene therapy applications.

One feature of *Sleeping Beauty* transposon system that makes it especially attractive in a wide variety of applications ranging from gene discovery to gene therapy is its near random integration capabilities. *SB* displays little preference for specific sequence flanking the target TA dinucleotide or for genomic region, such as genes versus intergenic regions [34–36]. From analysis of the current and limited *Tol2* integration site data, *Tol2* seems to be equally unbiased (Table 1 and [17]). A much larger sample similar to the ones analyzed for *SB* [34–36] will be required to know if *Tol2* exhibits any notable insertion-site preferences.

The ability to move large cargo opens new possibilities for applications in gene therapy, gene discovery, and transgenesis. For gene therapy, increased cargo-capacity accommodates large therapeutically relevant genes such as DNA-PKcs for X-linked severe combined immune deficiency [37] or factor VIII for hemophilia A [38]. This also allows for more complex vector design, including tissue-specific promoters, insulators, suicide genes, and other features that may improve both efficacy and safety of such vectors. In addition, lack of overexpression inhibition demonstrated by *Tol2* will be advantageous in designing *cis* vectors for co-delivery,of transposon and transposase, something that has required a great deal of optimization for *SB*. High cargo-capacity will facilitate adaptation of *Tol2* for gene discovery applications by allowing implementation of vectors that consist of multiple functional cassettes, for example, a 5′ gene-trap and a 3′ gene-trap, with different reporters. *Tol2* has been successfully adapted for enhancer identification in zebrafish [39], and the newly documented ability of *Tol2* to carry large cargos will facilitate such scanning of genomic sequences for regulatory elements. Animal biotechnology, an emerging area of transgenic research, will benefit from a large cargo-capacity vector that lacks overexpression inhibition.

*SB* and *Tol2* belong to different families of transposable elements: *SB* to the *Tc1/mariner* family and *Tol2* to the hAT family. These two highly active and very different transposon systems should have a strong synergistic effect in many areas of research. Different transgenes can be delivered to the same cell at the same or different time points without a concern of cross-mobilization. One such example is generation of transposase-expressing animals for cancer gene discovery. So far, transposase-expressing mice were generated either using plasmid transgenesis or by knock-in approaches [40–42]. It will now be possible to easily generate lines of animals that express SB transposase using *Tol2* transposons and vice versa.

Since the original publication of *Sleeping Beauty* transposon system [4], considerable research has been devoted to characterizing and improving transposition, and multiple laboratories have identified and published hyperactive variants of *SB* transposase [8,43–45]. Without any modifications, *Tol2* is already much more active than *SB* in zebrafish, but the activity of the two systems is comparable in mammalian cells. It remains to be determined if the *Tol2* hyperactivity potential seen in zebrafish can be mimicked in mammalian cells. Other members of the same hAT family to which *Tol2* belongs display additional beneficial characteristics that strongly suggest host factors are not necessary for transposition. Maize *Ac* is active in yeast [46] and *Drosophila Hermes* is active in vitro [16]. These observations suggest that *Tol2* can be further developed quite rapidly and thereby contribute insight into mechanisms of transposition as well as serve as a tool for many other biological applications.

## Materials and Methods

### Transposase RNA and DNA expression vectors.

The *Tol2* open reading frame was amplified by PCR using primers Tol2-F3 (5′-GCTGGATCCACCATGGAGGAAGTATGTGATTC-3′) and Tol2-R1 (5′-CAGACTAGTCTACTCAAAGTTGTAAAACC-3′) and the pBluescript-based Tol2 transposase vector described by Parinov et al. (2004) as template. The PCR fragment was first cloned into pCRIItopo vector (Invitrogen, Carlsbad, California, United States), resulting in pDB595. For RNA expression, the Tol2 ORF was subcloned from pDB595 as BamHI-SpeI fragment into BglII-SpeI digested transcription vector pT3TS [47], resulting in pT3TS-Tol2 (pDB600). pDB600 was cleaved with XbaI and transcribed with T3 polymerase using Ambion mMessage Machine kit (Austin, Texas, United States) to produce Tol2 mRNA. To make pCMV-Tol2, the transposase ORF was amplified from pDB595 using Tol2-NotI-F (5′-ATATGCGGCCGCCACCATGGAGGAAGTATG-3′) and M13R primers, cloned into pCR4topo vector (Invitrogen), and then a *Tol2* NotI fragment was excised and cloned into the NotI site of pCMV-bgal vector (Clontech, Palo Alto, California, United States) in the forward orientation. The SB10 ORF [4] was PCR subcloned into pT3TS to generate pT3TS-SB10.

### *Tol2* transposon vectors.

To construct pTol2/S2EF1a-GM2 (pDB591), the S2EF1a-GM2 expression cassette [13] was cloned as an SphI-NruI fragment of pDB371 between EcoRV sites of pGEM-T/Tol2 [11] in reverse orientation. To generate pTol2/SVneo (pDB625), the G418 resistance cassette of pT2/SVneo [8] was subcloned as HindIII fragment into EcoRV-digested pGemT-Tol2. To generate pminiTol2/SVneo (pDB678), the same fragment was cloned between HindIII and SwaI sites of pGEM-T/Tol2. The same fragment was cloned into the Eco105I site of pDB633 (see below) to make pTol2/FAHIL-SVneo (pDB691).

To define minimal sequences required for *Tol2* transposition in zebrafish somatic tissues, two strategies were used. *Tol2* sequences between SwaI and XcmI sites of pDB591 were deleted to make pDB665 (delta exon1), and sequences between XcmI and EcoO109I sites were deleted to make pDB664 (delta repeats). Also, S2EF1a-GM2 cassette was cloned into pGEM-T/Tol2 in reverse orientation between the following restriction enzyme sites: SwaI and EcoRV (pDB668, delta exon 1 and repeats), EcoRV and HindIII (pDB667, delta exon 4), and SwaI and HindIII (pDB674, *Tol2mini*). To make a “clipped” version of *Tol2* transposon, S2EF1a-GM2 expression cassette was amplified from pDB371 [13] using primers Tol2-sph-F (5′-CAGAGGTGTAAAGTACTTGAGTAATTTTACTTGATTACTGTACTTAAGTATGCATGCAAGCTAGTACAAGACG-3′) and Tol2-nru-R (5′-CAGAGGTGTAAAAAGTACTCAAAAATTTTACTCAAGTGAAAGTACAAGTACTTATCGCGATGATAATCAGCCATACC-3′). The PCR fragment was cloned into pCR4topo vector (Invitrogen) and sequenced.

To express FAH and firefly luciferase in mouse, bicistronic expression cassette consiting of miniCAGGS promoter, mouse FAH cDNA, encephalomyocarditis virus internal ribosome entry site, firefly luciferase, and rabbit β-globin polyadenylation sequences was subcloned as an NheI-PmeI fragment from pKT2/FAHIL (A. Wilber, K. J. Wangnesteen, Y. Chen, L. Zhuo, J. L. Frandsen, J. B. Bell, Z. J. Chen, S. C. Ekker, R. S. McIvor, and X. Wang, unpublished data) into EcoRV-digested pGEM-T/Tol2 [11] (pTol2/FAHIL) or SwaI-HindIII digested pGEM-T/Tol2 (pminiTol2/FAHIL).

### In vitro transposition assays*.*

A colony-forming assay was conducted as described [8]. Briefly, 3 × 10^5^ HT1080 cells were plated onto 6-cm plates and 24 h later were transfected with 1 to 2 μg of DNA using FuGene 6 reagent (Roche, Basel, Switzerland). Two days later, the cells were trypsinized and 3 × 10^4^ cells were plated onto 10-cm plates with complete medium supplemented with 600 μg/ml Geneticin (G418; Invitrogen). After 10 d of selection, the cells were stained with methylene blue and counted. Three plates of cells were transfected for each data point using different transposon DNA preps. For *Tol2/SB* activity comparison (Figure 2B), colonies were visually counted. For comparison of different-size *Tol2* elements (Figure 2C), colonies were counted using ImageJ software (http://rsb.info.nih.gov/ij). The number of colonies obtained in transfection including transposase was divided by the number of colonies obtained in control transfection to compute gene transfer efficiency. The efficiency of miniTol2/SVneo was set as 100%.

### Molecular analysis of genomic integration sites.

DNA was isolated from cultured cells or mouse liver using the Puregene DNA purification system (Gentra Systems, Minneapolis, Minnesota, United States). DNA was digested and used in an adapted inverse PCR (iPCR) protocol [12]. Tol2-specific iPCR primers were Tol2-R3 (5′-ACTGGGCATCAGCGCAATTCAATTG-3′) and Tol2-F7 (5′-AGCAGGATAAAACCTTGTATGCATT-3′) for the first round and Tol2-R4 (5′-ATAATACTTAAGTACAGTAATCAAG-3′) and Tol2-F8 (5′-CTCAAGTAAGATTCTAGCCAGATAC-3′) for the nested reaction. For iPCR of cell clones, Neo-resistant colonies were picked and amplified 2 wk after tranfection with pDB625 and pCMV-Tol2. Genomic DNA was extracted, digested with NheI, SpeI, and XbaI and ligated in a dilute reaction. The first round of iPCR was performed with Tol2-R3 and Tol2-F7, and a second with Tol2-R4 and Tol2-F7. The PCR products were isolated by gel electrophoresis, and sequenced using primers Tol2-R4 or Tol2-F7. For cloning of *miniTol2* insertion sites, HT1080 cells were transfected with miniTol2SVneo and pCMV-Tol2 and grown for 4 d without selection. Genomic DNA was extracted and digested with AflIII (which cuts the vector twice outside of the transposon and leaves incompatible ends) SpeI and XbaI, ligated in a dilute reaction, and one round of PCR was performed with Tol2-R3 and Tol2-F8. The resultant products were used in a Topo-cloning reaction (Invitrogen), and two insertions were identified by sequencing using M13-F and M13-R primers. For the mouse insertion sites, genomic DNA was extracted from each of five liver lobes of one FAH-deficient mouse 2 mo after injection with pTol2-FAHIL. The DNA was digested with NheI, BamHI, XhoI, and BsiWI, which do not cut vector sequences, blunted with Klenow fragment, and ligated in a dilute reaction. Two rounds of iPCR were performed and sequenced as described above for cell clones.

### Zebrafish somatic transposition assays*.*

For *SB*/*Tol2* comparison in the GFP fluorescence analysis (Figure 1), one-cell stage zebrafish embryos were injected with a mixture of 20 pg of pT2/S2EF1a/GM2 (pDB371) and pTol2/S2EF1a/GM2 (pDB591) each. Some of the embryos were then injected with 25 pg of T3TS-Tol2 or 25 pg of T3TS-SB RNA. Dead and grossly abnormal embryos were removed, and GFP fluorescence was scored at 3 d postfertilization. Randomly chosen embryos were photographed using Zeiss Axioscope II with Zeiss Axiocam using Zeiss Axiovision software. For excision analysis, embryos were injected with either a mixture of 20 pg of pT2/S2EF1a/GM2 (pDB371) DNA, 20 pg of pTol2/S2EF1a/GM2 (pDB591) DNA, and 25 pg of T3TS-Tol2 RNA or a mixture of 20 pg of pT2/S2EF1a/GM2 (pDB371) DNA, 20 pg of pTol2/S2EF1a/GM2 (pDB591) DNA, and 25 pg of T3TS-SB10 RNA. Twenty embryos were pooled at each indicated time point, and the DNA was prepared using a modified genomic DNA preparation protocol [48] and used for excision PCR [14]. To compare different Tol2 truncations, embryos were first injected with 25 pg of appropriate plasmid solution, and then all plasmid-injected embryos were injected with 25 pg of T3TS-Tol2 RNA using the same needle. Surviving and grossly normal embryos were scored for GFP fluorescence at 3 d postfertilization. Twenty GFP-positive embryos were used for DNA preparation. DNA was dissolved in 50 μl of TE and diluted 1:1 in water; 1 μl of this solution was subsequently used for excision PCR (see above).

### Animals and plasmid injections.

Normal C57BL/6 mice and FAHDexon 5 transgenic (tg) mice [24], 18 to 25 g, were bred in a specific pathogen-free environment at the University of Minnesota. FAH-deficient mice were maintained on drinking water supplemented with NTBC at a concentration of 7.5 mg/ml. Plasmids were delivered primarily to the liver using a rapid, high-volume tail vein injection procedure [21,22] as previously described [49]. Only animals that received a complete injection in less than 8 s were used for subsequent studies.

### In vivo bioluminescent imaging.

At various times after injection of DNA, animals were anesthetized by intraperitoneal injection of 160 mg/kg ketamine plus 0.8 mg/kg acepromazine and 0.08 mg/kg butorphanol. One hundred milliliters of 28.5 mg/ml luciferin substrate was then injected intraperitoneally. At 4 to 5 min after injection of substrate, the live anesthetized mice were imaged for 1 s to 2 min using an intensified, charge-coupled device camera (Series 100; Xenogen, Hopkinton, Massachusetts, United States) as described [49]. Raw values of luciferase activity were recorded as photons of light emitted per second.

## Abbreviations

FAH: fumarylacetoacetate hydrolase
GFP: green fluorescent protein
HT1: hereditary tyrosinemia type 1
NTBC: 2-(2-nitro-4-trifluoro-methylbenzoyl)-1,3 cyclohexanedione
